# Properties and interactions – melting point of tri­bromo­benzene isomers

**DOI:** 10.1107/S2052520621006399

**Published:** 2021-07-24

**Authors:** Maciej Bujak, Marcin Podsiadło, Andrzej Katrusiak

**Affiliations:** a Faculty of Chemistry, University of Opole, Oleska 48, Opole, 45-052, Poland; b Faculty of Chemistry, Adam Mickiewicz University, Uniwersytetu Poznańskiego 8, Poznań, 61-614, Poland

**Keywords:** tribromobenzene isomers, structure-property relationship, melting point, molecular symmetry, noncovalent interactions, halogen bond

## Abstract

Single crystals of isomeric 1,2,3-tri­bromo­benzene (123TBB), 1,2,4-tri­bromo­benzene (124TBB) and 1,3,5-tri­bromo­benzene (135TBB) have been grown from different solvents and their structures determined by X-ray diffraction at 100, 200 and 270 K. The melting-point differences of *ca* 40 K between 135TBB, 123TBB and 124TBB have been correlated with the molecular symmetry and packing preferences in the crystal, as well as with the main types of intermolecular halogen interactions, *i.e.* Br⋯Br, Br⋯C (Br⋯π) and Br⋯H. The relationship between symmetry and melting point in Carnelley’s rule has been extended to the accessibility of terminal atoms for the formation of intermolecular interactions, their occurrences and distribution, and the close packing. The electrostatic potential mapped on molecular surfaces demonstrates that in more symmetric molecules the more evenly distributed substituents are more accessible and form more optimum intermolecular interactions.

## Abstract 2

The melting points of tri­bromo­benzene isomers are correlated with the number, nature and distribution of intermolecular interactions in their structures.

## 1. Introduction   

Melting point is one of the fundamental thermodynamic parameters that can be quickly and cheaply determined, and used, along with other parameters, to identify a substance, check its purity and characterize its properties. Most physicochemical tables list melting and boiling points, and solubility, along with other parameters of various compounds. These parameters change in a characteristic way for compounds forming a homologous series, for example, analogous derivatives with different substituents. However, the most interesting from a structural point of view are the exceptions and anomalies in the well established trends of these series, and in particular polymorphs and very similar compounds, including isomers.

Numerous studies have been dedicated to the rules and factors affecting the melting point that could be calculated as the quotient of enthalpy and entropy of melting (Carnelley, 1882 ▸; Beacall, 1928 ▸; Holler, 1948 ▸; Gavezzotti, 1995 ▸; Brown & Brown, 2000 ▸; Boese *et al.*, 1999 ▸; Thalladi & Boese, 2000 ▸; Thalladi *et al.*, 2000*a* ▸,*b* ▸,*c* ▸; Katritzky *et al.*, 2001 ▸; Bujak *et al.*, 2008 ▸; Joseph *et al.*, 2011 ▸; Podsiadło *et al.*, 2012 ▸, 2013 ▸; Yalkowsky & Alantary, 2018 ▸; Gallagher *et al.*, 2019 ▸). The studies showed that the location of substituents in molecules and their ability to form intermolecular interactions cannot be neglected when explaining the melting-point differences of isomers. In a recent study on tetra­chloro­benzene isomers, both the nature and distribution of interactions were correlated with the melting points of these relatively simple compounds, by representing the molecular symmetry and magnitudes of electrostatic potential on the molecular surfaces (Bujak, 2018 ▸).

In this article, we continue our studies on the differences in melting points of isomers for tri­bromo­benzenes (Scheme 1 shows the tri­bromo­benzene isomers, their abbreviations, the molecular point-group symmetries and the melting points). We have determined the crystal structures at 270, 200 and 100 K. The experimental study is supported by a Hirshfeld surface analysis and quantum-chemical calculations. We were particularly interested in the nature of the intermolecular interactions, their thermal behaviour and the relationships of the various structural parameters to the differences in the melting points of these compounds. 
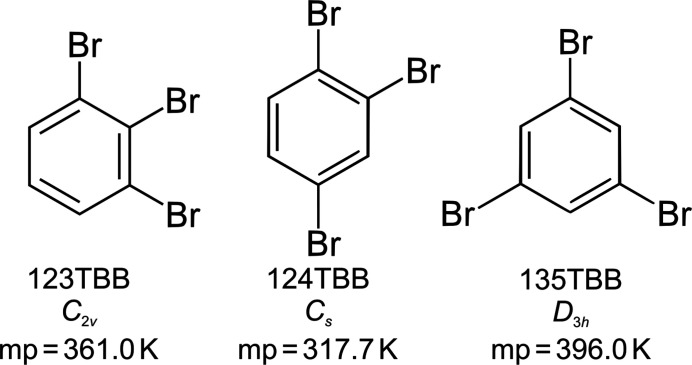


**Figure 1:**
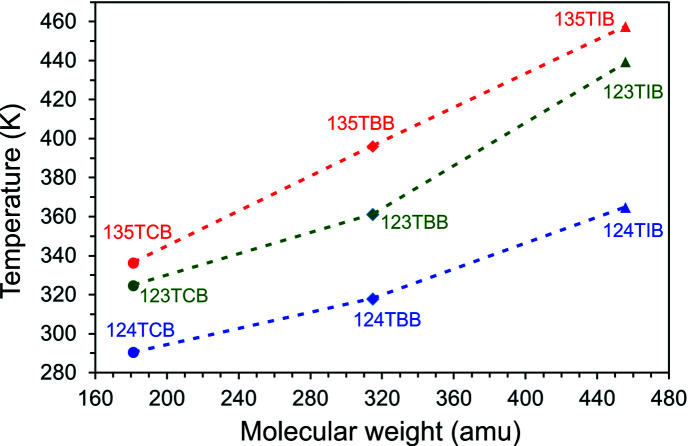
Melting points (Mackay *et al.*, 2006 ▸) for series of isomers: (•) tri­chloro- (TCB), (♦) tri­bromo- (TBB) and (▴) tri­iodo­benzenes (TIB), plotted as a function of their molecular weight. The dashed lines joining the points are guides for the eye only.

All tri­bromo­benzene isomers are solid under ambient conditions, with their melting points above 315 K (Mackay *et al.*, 2006 ▸). As illustrated in Fig. 1 ▸, in accordance with the molecular symmetry and Carnelley’s rule (Carnelley, 1882 ▸; Brown & Brown, 2000 ▸), the highest melting point is that of 135TBB (396.0 K, molecular symmetry *D*_3*h*_), then lower by 35 K is that of 123TBB (361.0 K *C*_2*v*_ symmetry) and still 43 K lower is that of 124TBB (317.7 K, *C* *_s_* symmetry). These melting-point differences indicate that the structural dissimilarities between the tri­bromo­benzene isomers could be related to their molecular symmetry (*i.e.* the location of the Br atoms in the aromatic ring), as well as the distinct molecular interactions and crystal packing preferences.

Of all the tri­bromo­benzenes, so far only the structure of 135TBB was determined at room temperature (Milledge & Pant, 1960 ▸; Belaaraj *et al.*, 1984 ▸). Milledge & Pant (1960 ▸) established that 135TBB does not undergo any discontinuous phase transition between room and liquid-nitro­gen temperature.

## 2. Experimental   

Samples of 123TBB (98%; Tokyo Chemical Industry Co. Ltd), 124TBB and 135TBB (95 and 99%, respectively; Fluoro­chem Ltd) were recrystallized from different solvents. The best crystals of 123TBB and 124TBB were obtained at room temperature by the slow evaporation from ethanol (96%, POCH S.A.) solutions, whereas for 135TCB, the tetra­chloro­methane (EUROCHEM BGD Sp. z o.o.) solution performed better.

Single crystals of the tri­bromo­benzene isomers were selected under a polarizing microscope and sealed in thin-walled glass capillaries (from Hilgenberg GmbH, with an internal diameter of 0.3 mm and walls 0.01 mm thick). The samples were cooled in a stream of nitro­gen gas from an Oxford Cryosystems attachment. The diffraction data were collected for each crystal first at 270 K and then at 200 K and 100 K on a single-crystal Xcalibur Eos CCD diffractometer with graphite-monochromated Mo *K*α radiation. Reflections were measured using the ω-scan technique with Δω = 1.0° images exposed for 10 (for 123TBB and 135TBB) and 7.5 s (for 124TBB). *CrysAlisPro* (Rigaku Oxford Diffraction, 2018 ▸) was used for collecting data and data reduction. All data were accounted for Lorentz, polarization and analytical sample absorption effects. The structures were solved by direct methods and refined with *SHELX* (Sheldrick, 2008 ▸, 2015 ▸). Noncentrosymmetric 124TBB and 135TBB were refined as inversion twins. The H-atom positions in all the structures were located in difference Fourier maps and the riding model was applied. The isotropic displacement parameters of the H atoms were fixed to 1.2*U*_eq_ of their carriers.

Intermolecular contacts were compared using Hirshfeld surface analysis with *CrystalExplorer17* (Turner *et al.*, 2017 ▸) and the structures were drawn with *Mercury* (Macrae *et al.*, 2020 ▸). The geometry optimizations and the calculations for 123TBB, 124TBB and 135TBB were performed using the *Gaussian 09* program package (Frisch *et al.*, 2009 ▸), along with the *GaussView 5.0* graphical interface (Dennington *et al.*, 2009 ▸). Density functional theory (DFT) calculations were carried out at the B3LYP/6-311-G(2d,2p) level of theory (Becke, 1988 ▸; Lee *et al.*, 1988 ▸), in a vacuum, for the starting models of the isolated molecules adopted from the X-ray diffraction results.

## 3. Results   

123TBB crystallizes in the monoclinic space group *P*2_1_/*c*, 124TBB in the orthorhombic space group *Fdd2* and 135TBB in the orthorhombic space group *P*2_1_2_1_2_1_. There is one independent molecule in the asymmetric unit for 135TBB, two for 123TBB and three for 124TBB. For these isomers, no transition or symmetry changes were detected from 270 to 100 K. In this temperature range, the volume of the unit cells of the crystals similarly contract by 3.01 (123TBB), 2.67 (124TBB) and 3.04% (135TBB); their unit-cell parameters contract nearly linearly, but at different rates. The largest linear contraction of 1.49% is that of the shortest *a* parameter in 135TBB (Table 1 ▸ and Tables S1–S3 in the supporting information).

The crystals of all the studied isomers, similar to other halogenated benzenes, can be considered as being built of layers composed of tri­bromo­benzene molecules connected through Br⋯Br/C/H interactions (Figs. S1–S3 in the supporting information). At 100 K, the distance between parallel aromatic rings is similar, *ca* 3.5 Å, in both 123TBB and 135TBB, and it is *ca* 3.6 Å in 124TBB. The molecules of 123TBB, 124TBB and 135TBB are nearly planar as scrutinized in the supporting information.

The molecular dimensions of the studied tri­bromo­benzenes show some steric hindrance between adjacent Br and H substituents in the aromatic rings. The shortest intramolecular distances of Br⋯Br = 3.307 (1) Å, H⋯H = 2.30 Å and Br⋯H = 2.83 Å, which are clearly below the sums of the van der Waals radii (Bondi, 1964 ▸), are present in 123TBB and 124TBB at 270 K.

Intermolecular contacts involving Br atoms are present in all isomers and their lengths decrease on cooling (Table 2 ▸ and Tables S7, S8 and S10). The largest contraction of −0.081 (5) Å between 270 and 100 K is for the Br11⋯Br34 halogen bond in 124TBB. Also, the Br⋯H contacts, in general, contract on cooling (Tables S8 and S9). Interestingly, according to the criterion of the sum of the van der Waals radii, at 270 K, intermolecular Br⋯Br bonds are present only in 123TBB and 124TBB, whereas the first Br⋯Br halogen bond in 135TBB appears in the structure at *ca* 175 K (Tables 2 ▸ and S10). The intermolecular contacts for the structures of the isomers at 100 K are discussed below.

In 123TBB, the intermolecular Br11⋯Br12 distance is 3.5850 (6) Å. Atoms Br13 and Br23 are involved in C13—Br13⋯π and C23—Br23⋯π interactions (Pang *et al.*, 2013 ▸; Wang *et al.*, 2016 ▸; Mahadevi & Sastry, 2016 ▸). The shortest Br⋯C separations are Br13⋯C23 of 3.393 (4) Å and Br23⋯C13 of 3.395 (5) Å, whereas the corresponding Br⋯π(centroid) distances are 3.392 (2) and 3.464 (2) Å, respectively (Spek, 2020 ▸; Tables 2 ▸ and S7, and Fig. S4). The intermolecular interactions of the 124TBB molecules are different. According to the criterion of the van der Waals radii, none of the independent molecules form C—Br⋯π interactions, but only Br⋯Br and Br⋯H interactions. Molecule C(11–C16) is involved in six interactions, while molecules C(21–26) and C(31–36) are involved in four halogen bonds each. The shortest Br⋯Br and Br⋯H distances are 3.565 (3) and 2.97 Å, respectively (Tables 2 ▸, S8 and S9, and Fig. S5). In 135TBB, the Br13⋯Br15 distance is 3.6994 (7) Å and atom Br13 is also engaged in two Br⋯Br bonds of 3.6724 (8) Å, as well as in one Br13⋯π interaction, characterized by Br⋯C distance of 3.549 (5) Å and a Br⋯π(centroid) distance of 3.786 (2) Å (Spek, 2020 ▸; Tables 2 ▸ and S10, and Fig. S6).

The contribution of specific types of interactions to the overall cohesion forces in the crystals can be estimated roughly by the number and interatomic distances of the intermolecular contacts for one molecule. According to this criterion, the largest contribution of the Br⋯Br bonds is in 135TBB. In this compound, the Br atoms are more exposed and better accessed compared to 123TBB and 124TBB. At low temperature, due to crystal contraction, the distances become shorter, approximately at the same rate for all isomers.

The Br⋯Br halogen bonds formed in tri­bromo­benzenes are somewhat distorted from idealized types I and II. In both 124TBB and 135TBB, the Br⋯Br interactions, mainly of electrostatic type II, are relatively long. In contrast, the 123TBB crystal structure is governed by relatively short dispersion type I Br⋯Br bonds (Desiraju & Parthasarathy, 1989 ▸; Pedireddi *et al.*, 1994 ▸; Awwadi *et al.*, 2006 ▸; Fourmigué, 2009 ▸; Mukherjee *et al.*, 2014 ▸; Cavallo *et al.*, 2016 ▸).

**Figure 2:**
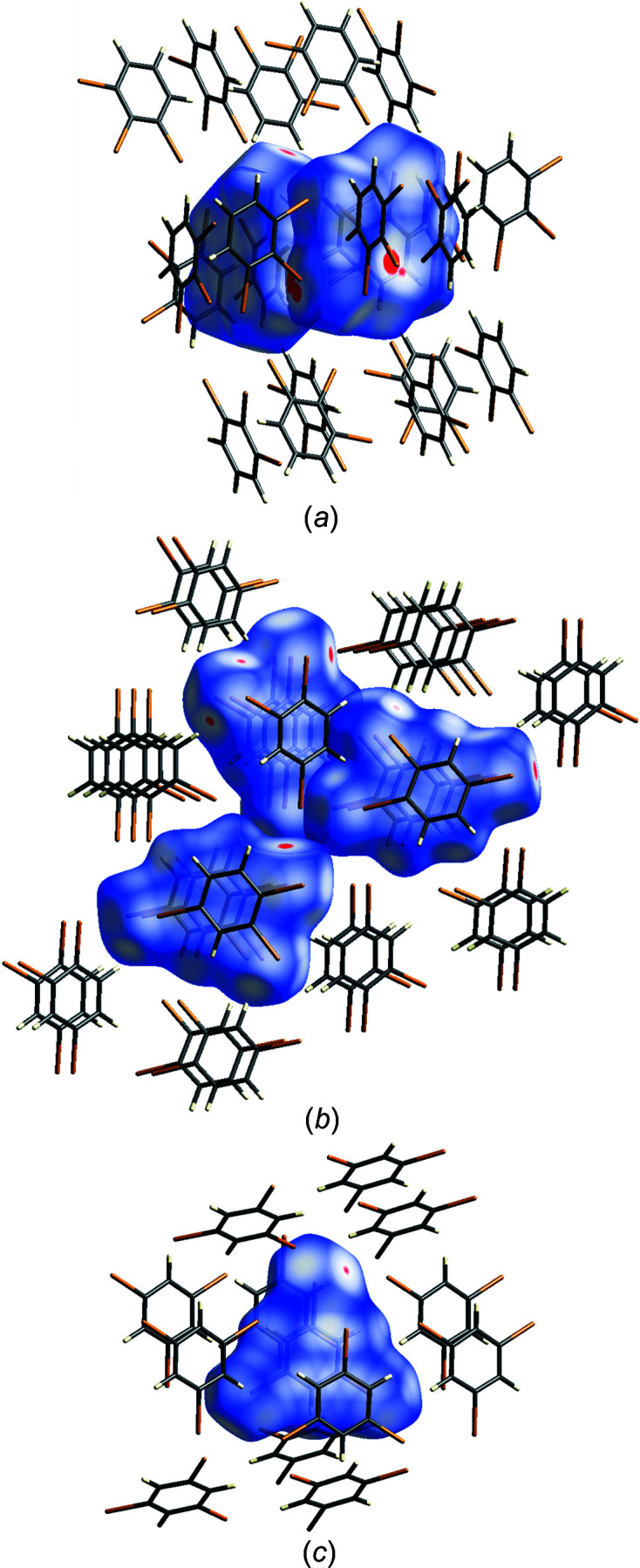
Hirshfeld surfaces (generated separately for symmetry-independent molecules) mapped with *d*_norm_ (−0.02 to 0.80) and close molecules within a radius of 4.0 Å, at 100 K, for (*a*) two 123TBB molecules [C(11–16) left and C(21–26) right]; (*b*) three 124TBB molecules [C(11–16) upper, C(21–26) middle and C(31–36) bottom] and (*c*) 135TBB.

The Hirshfeld surfaces and corresponding fingerprint plots illustrate the distribution of the types of cohesion forces in tri­bromo­benzenes (Turner *et al.*, 2017 ▸; McKinnon *et al.*, 2004 ▸, 2007 ▸; Spackman & Jayatilaka, 2009 ▸). This analysis covers a broad range of possible intermolecular contacts, also those longer than the sums of the van der Waals radii. Contacts shorter than the sums of the van der Waals radii, coloured red in Figs. 2 ▸ and S7, mainly relate to Br⋯Br, Br⋯C (Br⋯π) and Br⋯H. All the fingerprint plots contain the sharp central red regions representing Br⋯Br and two symmetrically located blue spikes for Br⋯H contacts. The blue regions close to the Br⋯Br red areas are due to Br⋯C (Br⋯π) contacts (Fig. S7*c* ). The plot for molecule C(11–16) of 124TBB has a different distribution of H⋯H contacts (blue area between the Br⋯H spikes; Fig. S7*b* ).

## 4. Discussion   

**Figure 3:**
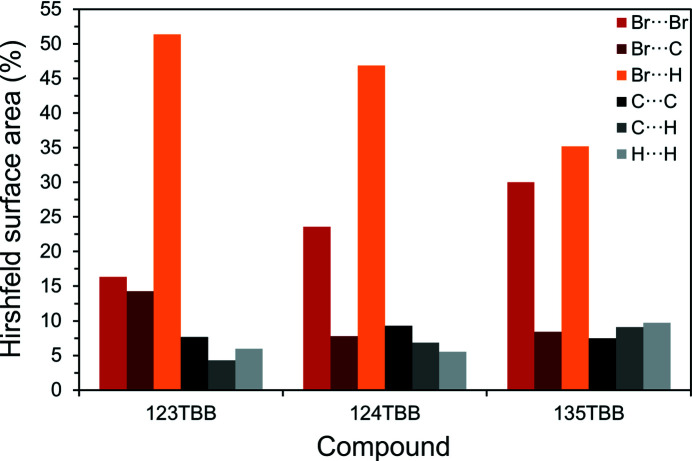
Distribution of contacts, at 100 K, based on their area on the Hirshfeld surfaces for 123TBB, 124TBB and 135TBB (the averaged values for the independent molecules are presented, *cf.* Fig. S8).

Because in all the TBB isomers the high and low magnitudes of the calculated electrostatic potentials are associated with the Br atoms, one can expect that the electrostatic interactions mainly govern the aggregation of molecules. Fig. 3 ▸ shows the contribution of different contacts, assessed according to their portion on the Hirshfeld surfaces. The contacts involving Br atoms, *i.e.* Br⋯Br, Br⋯C (Br⋯π) and Br⋯H, constitute at least *ca* 74% of all contacts in the three isomers. It appears from these statistics that the combined contacts of Br⋯Br and Br⋯C (Br⋯π) correlate with the highest melting point of 135TBB; the sum of the Br⋯Br and Br⋯C (Br⋯π) contributions is *ca* 38 *versus* *ca* 31% for both 123TBB and 124TBB. At the same time, the sum of the Br⋯C (Br⋯π) and Br⋯H contacts for 123TBB (*ca* 66%) is clearly higher than that for the less symmetric isomer 124TBB (*ca* 55%), having the lowest melting point.

Neither the variances in the contribution of specific types of contacts at different temperatures nor those for independent molecules significantly affect the above-mentioned analysis (Fig. S8). Furthermore, as mentioned previously, the Br⋯Br halogen bonds in both 135TBB and 124TBB are of distorted type II, whereas those in 123TBB are of distorted type I. Also, the shortest Br⋯H bonds, found in 124TBB only, are of both distorted type I and II interactions. This shows that both the nature and distribution of specific cohesion forces may contribute to the differentiation of the melting points of isomeric tri­bromo­benzenes.

The magnitudes of the calculated molecular electrostatic potential are correlated with the preferences of particular atoms to form intermolecular interactions (Cavallo *et al.*, 2016 ▸; Fig. S9). The electrostatic potential on the Br atoms in 135TBB is equally distributed, which favours electrostatic type II halogen bonds evenly distributed in space, because Br atoms are easily accessed for intermolecular interactions.

From a thermodynamic point of view, melting is a process that depends on the balance between the enthalpy and entropy of melting. The enthalpy component is associated mainly with molecular size and intermolecular interactions, whereas entropy, related to molecular symmetry and flexibility, affects the arrangement and packing of molecules (Yalkowsky & Alantary, 2018 ▸). Therefore, the high symmetry of molecules facilitates their interactions and crystal formation, which results in a lower entropy of melting and in a higher melting point.

**Figure 4:**
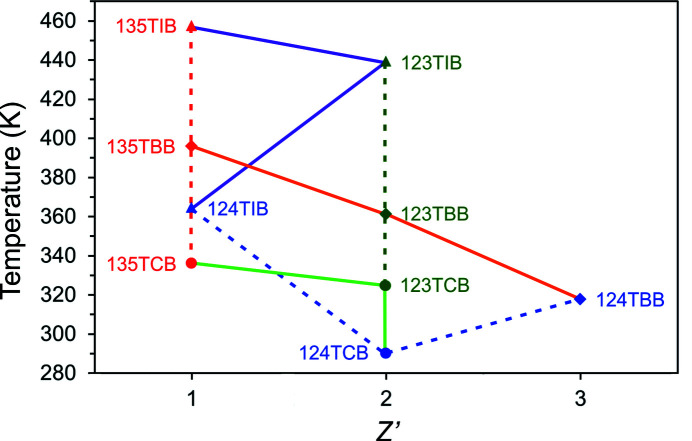
Melting points for series of isomers: (•) tri­chloro- (TCB), (♦) tri­bromo- (TBB) and (▴) tri­iodo­benzenes (TIB), plotted as a function of *Z*′. The solid lines join the points for isomers containing the same halogen atoms, while the dashed lines are for isomers with the same halogen-atom positions in an aromatic ring. Both the solid and dashed lines are guides for the eye only.

The selected structural and thermodynamic parameters for the tri­bromo­benzene isomers are collected in Table 3 ▸. The unit-cell volume per molecule (*V*/*Z*) and the calculated density parameters are comparable for all the studied isomers. The void volume/*Z* and the relative volume of voids gradually decreases from 123TBB to 124TBB and are lowest for 135TBB (Figs. S1–S3). It appears that the melting point for tri­bromo­benzenes decreases with an increasing number of molecules in the asymmetric unit (*Z*′); however, this regularity does not apply to the 124TIB and 124TCB isomers (Fig. 4 ▸). It is intriguing that in this series of isomers, the least dense 135TBB has the highest melting point, which is inconsistent with the observation that more dense crystals usually have higher melting points.

The tri­bromo­benzene isomers are composed of ordered rigid molecules; thus, the differences in their melting points, like those of disubstituted benzenes (Gavezzotti, 1995 ▸; Bujak *et al.*, 2007 ▸; Dziubek & Katrusiak, 2014 ▸), can be associated with the enthalpy-related components, such as cohesion forces. This further confirms the arguments based on the analysis of the nature and distribution of intermolecular interactions.

## 5. Conclusions   

The crystals of three tri­bromo­benzene isomers have been investigated at low temperature in order to explore the relationships between the intermolecular interactions and melting points of these compounds. Beside the symmetry, described by Carnelley’s rule, the intuitive principle for the melting-point differences can be associated with cohesion forces: the stronger and more frequent the interactions between molecules the more energy is required to melt the crystal. The present results show that the strongest halogen bonds alone cannot account for the melting-point differences. Therefore weaker intermolecular interactions have to be considered too. Higher molecular symmetry provides better access to the Br atoms and hence the specific optimum interactions that correlate with the higher melting point of the particular isomer.

## Supplementary Material

Crystal structure: contains datablock(s) global, 123TBB_270K, 123TBB_200K, 123TBB_100K, 124TBB_270K, 124TBB_200K, 124TBB_100K, 135TBB_270K, 135TBB_200K, 135TBB_100K. DOI: 10.1107/S2052520621006399/lo5092sup1.cif

Structure factors: contains datablock(s) 123TBB_270K. DOI: 10.1107/S2052520621006399/lo5092123TBB_270Ksup2.hkl

Structure factors: contains datablock(s) 123TBB_200K. DOI: 10.1107/S2052520621006399/lo5092123TBB_200Ksup3.hkl

Structure factors: contains datablock(s) 123TBB_100K. DOI: 10.1107/S2052520621006399/lo5092123TBB_100Ksup4.hkl

Structure factors: contains datablock(s) 124TBB_270K. DOI: 10.1107/S2052520621006399/lo5092124TBB_270Ksup5.hkl

Structure factors: contains datablock(s) 124TBB_200K. DOI: 10.1107/S2052520621006399/lo5092124TBB_200Ksup6.hkl

Structure factors: contains datablock(s) 124TBB_100K. DOI: 10.1107/S2052520621006399/lo5092124TBB_100Ksup7.hkl

Structure factors: contains datablock(s) 135TBB_270K. DOI: 10.1107/S2052520621006399/lo5092135TBB_270Ksup8.hkl

Structure factors: contains datablock(s) 135TBB_200K. DOI: 10.1107/S2052520621006399/lo5092135TBB_200Ksup9.hkl

Structure factors: contains datablock(s) 135TBB_100K. DOI: 10.1107/S2052520621006399/lo5092135TBB_100Ksup10.hkl

Additional Tables and Figures. DOI: 10.1107/S2052520621006399/lo5092sup11.pdf

CCDC references: 2041515, 2041516, 2041517, 2041518, 2041519, 2041520, 2041521, 2041522, 2041523

## Figures and Tables

**Table 1:** Selected crystal data for 123TBB, 124TBB and 135TBB at 100 K

	123TBB	124TBB	135TBB
Chemical formula	C_6_H_3_Br_3_	C_6_H_3_Br_3_	C_6_H_3_Br_3_
*M*_r_	314.78	314.78	314.78
Crystal system	Monoclinic	Orthorhombic	Orthorhombic
Space group, *Z*, *Z*′	*P*2_1_/*c*, 8, 2	*Fdd*2, 48, 3	*P*2_1_2_1_2_1_, 4, 1
*a* (Å)	12.7973 (5)	29.313 (2)	4.00341 (11)
*b* (Å)	8.2623 (3)	78.645 (4)	13.4119 (3)
*c* (Å)	15.4666 (6)	3.9320 (2)	14.0916 (4)
β (°)	113.102 (5)	90	90
*V* (Å^3^)	1504.22 (11)	9064.5 (9)	756.63 (3)
*R*[*F* ^2^ > 2σ(*F* ^2^)]	0.027	0.061	0.018
*wR*(*F* ^2^)	0.055	0.135	0.036

**Table 2:** Short intermolecular Br⋯Br distances (Å) and angles (°) in 123TBB, 124TBB and 135TBB at 270, 200 and 100 K

	270 K	200 K	100 K
123TBB			
Br11⋯Br12^i^	3.6440 (10)	3.6175 (8)	3.5850 (6)
C11—Br11⋯Br12^i^	162.75 (19)	162.83 (17)	163.37 (15)
Br11⋯Br12^i^—C12^i^	135.03 (16)	135.19 (14)	135.10 (12)
			
124TBB			
Br11⋯Br34^ii^	3.687 (4)	3.651 (3)	3.606 (3)
C11—Br11⋯Br34^ii^	99.2 (7)	99.4 (6)	99.7 (5)
Br11⋯Br34^ii^—C34^ii^	171.2 (7)	172.6 (6)	172.2 (6)
Br14⋯Br21^iii^	3.751 (4)	3.722 (3)	3.676 (3)
C14—Br14⋯Br21^iii^	86.3 (6)	86.2 (6)	86.0 (6)
Br14⋯Br21^iii^—C21^iii^	161.4 (7)	162.0 (6)	162.1 (6)
Br14⋯Br31	3.698 (4)	3.672 (3)	3.640 (3)
C14—Br14⋯Br31	165.7 (8)	165.2 (7)	165.4 (6)
Br14⋯Br31—C31	105.6 (7)	105.0 (6)	105.5 (6)
Br21⋯Br24^iv^	3.630 (4)	3.606 (3)	3.565 (3)
C21—Br21⋯Br24^iv^	102.7 (6)	102.3 (6)	103.3 (5)
Br21⋯Br24^iv^—C24^iv^	174.2 (7)	174.9 (6)	175.9 (6)
			
135TBB			
Br13⋯Br13^v^	3.7411 (13)	3.7094 (9)	3.6724 (8)
C13—Br13⋯Br13^v^	151.9 (2)	152.15 (16)	152.14 (15)
Br13⋯Br13^v^—C13^v^	112.4 (2)	112.16 (15)	112.30 (14)
Br13⋯Br15^vi^	3.7458 (11)	3.7252 (8)	3.6994 (7)
C13—Br13⋯Br15^vi^	118.4 (2)	117.95 (15)	117.93 (15)
Br13⋯Br15^vi^—C15^vi^	156.1 (2)	155.94 (17)	156.24 (16)

Symmetry codes: (i) −*x* + 1, *y* + 1 \over 2, −*z* + 1 \over 2; (ii) *x* − 1 \over 2, *y*, *z* + 3 \over 2; (iii) *x*, *y*, *z* + 1; (iv) *x* + 1 \over 4, −*y* + 1 \over 4, *z* − 3 \over 4; (v) *x* − 1 \over 2, −*y* + 1 \over 2, −*z*; (vi) −*x* + 1 \over 2, −*y* + 1, *z* − 1 \over 2.

**Table 3:** Comparison of selected structural and thermodynamic parameters for 123TBB, 124TBB and 135TBB at 100 K

	123TBB	124TBB	135TBB
Space group	*P*2_1_/*c*	*Fdd*2	*P*2_1_2_1_2_1_
*Z*, *Z*′	8, 2	48, 3	4, 1
*V*/*Z*	188.03	188.8	189.16
Density (calculated, g cm^−3^)	2.780	2.768	2.763
Void volume/*Z* (Å^3^)	16.2	10.3	7.4
Void volume of *V* (%)	8.6	5.4	3.9
Molecular symmetry	*mm*2; *C*_2*v*_	*m*; *C_s_*	\overline 6*m*2; *D*_3*h*_
Boiling point (K)	556*	548	544
Melting point (K)	361.0	317.7	396.0
Enthalpy of melting (kJ mol^−1^)	Not available	17.9	21.7
Entropy of melting (J K^−1^ mol^−1^)	Not available	56**	55**

Notes and references: (*) Nakada *et al.* (1970 ▸); (**) entropy of melting calculated from the enthalpy of melting (Kuramochi *et al.*, 2004 ▸; van der Linde *et al.*, 2005 ▸) and the melting point (Mackay *et al.*, 2006 ▸).
