# Supplementary material for: Properties and interactions – melting point of tri­bromo­benzene isomers
Source: Acta Crystallogr B Struct Sci Cryst Eng Mater. 2021 Jul 24;77(Pt 4):632–7. doi: 10.1107/S2052520621006399 (PMC8500239; doi:10.1107/S2052520621006399)
Supplement: Supplementary file 11 [file b-77-00632-sup11.pdf]

STRUCTURAL SCIENCE
CRYSTAL ENGINEERING
MATERIALS

Volume 77 (2021)

Supporting information for article:

Properties and interactions – melting point of tribromobenzene isomers

Maciej Bujak, Marcin Podsiadło and Andrzej Katrusiak

Accuracy of structural determinations (SI)

At 100 K, the maximum deviations of the Br and C atoms from the nine-atom least squares planes are 0.018(2) Å for 135TBB, 0.024(4) Å for two independent molecules of 123TBB; and 0.056(8) Å for three independent molecules of 124TBB. The C–C bond lengths, at 100 K, of average values 1.385 Å for 123TBB, 124TBB and 135TBB, are consistent with those in the structure of solid benzene determined at 100 K (average 1.395 Å, Woińska *et al*., 2016). The mean C–Br length of 1.893 Å for all tribromobenzene isomers is comparable to that found in 135TBB at room temperature (Milledge & Pant, 1960) as well as in other polybromobenzenes (Reddy *et al.*, 2006; Dziubek & Katrusiak, 2014; Saha & Desiraju, 2017; Zakharov *et al.*, 2018). The C–C–C aromatic angles are as expected (Woińska *et al.*, 2016, Tables S4-S6). The above mentioned bond lengths and angles are also analogous to previously measured dimensions for 135TBB by electron diffraction (de Laszlo, 1934; Kossiakoff & Springall, 1941; Novikov *et al.*, 1986).

Table S1 Selected low-temperature crystal data and structure determination summary for 123TBB at 270, 200 and 100 K.

temperature (K)	270.0(1)	200.0(1)	100.0(1)
formula	$C_6H_3Br_3$	$C_6H_3Br_3$	$C_6H_3Br_3$
M_r	314.78	314.78	314.78
crystal size (mm)	0.48×0.25×0.18	0.48×0.25×0.18	0.48×0.25×0.18
crystal system	monoclinic	monoclinic	monoclinic
space group, Z, Z'	$P2_1/c$, 8, 2	$P2_1/c$, 8, 2	$P2_1/c$, 8, 2
a (Å)	12.9832(8)	12.8999(7)	12.7973(5)
b (Å)	8.3806(6)	8.3264(5)	8.2623(3)
c (Å)	15.5159(10)	15.4925(8)	15.4666(6)
β (deg)	113.276(7)	113.204(6)	113.102(5)
V (Å^3)	1550.8(2)	1529.44(16)	1504.22(11)
ρ (g/cm^3)	2.697	2.734	2.780
μ (mm^{-1})	15.512	15.729	15.993
θ range (deg)	2.86 - 26.00	2.86 - 26.00	2.85 - 26.00
index ranges	$-16 \le h \le 16$	$-15 \le h \le 15$	$-15 \le h \le 15$
	$-10 \le k \le 10$	$-10 \le k \le 10$	$-10 \le k \le 10$
	$-19 \le l \le 19$	$-19 \le l \le 19$	$-19 \le l \le 19$
reflns collected	21557	21168	20735
R_{int}	0.0862	0.0721	0.0595
data [$I > 2\sigma(I)$]	1880	2138	2364
data/parameters	3041/164	2990/164	2944/164
GOF on F^2	0.976	1.003	1.043
R_1 [$I > 2\sigma(I)$]	0.0395	0.0349	0.0271
R_1 (all data)	0.0862	0.0623	0.0420
wR_2 (all data)	0.0753	0.0676	0.0545
lrgst diff peak (e/Å^3)	0.630	0.718	0.706
lrgst diff hole (e/Å^3)	–0.687	–0.679	–0.587
CCDC	2041521	2041522	2041523

Table S2 Selected low-temperature crystal data and structure determination summary for 124TBB at 270, 200 and 100 K.

temperature (K)	270.0(1)	200.0(1)	100.0(1)
formula	$C_6H_3Br_3$	$C_6H_3Br_3$	$C_6H_3Br_3$
M_r	314.78	314.78	314.78
crystal size (mm)	0.93×0.13×0.07	0.93×0.13×0.07	0.93×0.13×0.07
crystal system	orthorhombic	orthorhombic	orthorhombic
space group, Z, Z'	*Fdd2*, 48, 3	*Fdd2*, 48, 3	*Fdd2*, 48, 3
a (Å)	29.408(3)	29.383(2)	29.313(2)
b (Å)	79.439(6)	79.163(4)	78.645(4)
c (Å)	3.9865(4)	3.9713(3)	3.9320(2)
V (Å^3)	9313.0(15)	9237.4(10)	9064.5(9)
ρ (g/cm^3)	2.694	2.716	2.768
μ (mm^{-1})	15.499	15.626	15.924
θ range (deg)	2.77 - 26.00	2.77 - 26.00	2.78 - 26.00
index ranges	$-36 \le h \le 36$	$-36 \le h \le 36$	$-36 \le h \le 36$
	$-83 \le k \le 97$	$-83 \le k \le 97$	$-82 \le k \le 97$
	$-4 \le l \le 4$	$-4 \le l \le 4$	$-4 \le l \le 4$
reflns collected	19561	19496	19208
R_{int}	0.1387	0.1155	0.1027
data [$I > 2\sigma(I)$]	2161	2801	3365
data/parameters	4476/245	4444/245	4368/245
GOF on F^2	0.948	1.004	1.069
R_1 [$I > 2\sigma(I)$]	0.0672	0.0621	0.0613
R_1 (all data)	0.1525	0.1080	0.0863
wR_2 (all data)	0.1604	0.1373	0.1348
lrgst diff peak (e/Å^3)	1.325	1.753	2.179
lrgst diff hole (e/Å^3)	–0.843	–0.896	–1.717
CCDC	2041518	2041519	2041520

Table S3 Selected low-temperature crystal data and structure determination summary for 135TBB at 270, 200 and 100 K.

temperature (K)	270.0(1)	200.0(1)	100.0(1)
formula	$C_6H_3Br_3$	$C_6H_3Br_3$	$C_6H_3Br_3$
M_r	314.78	314.78	314.78
crystal size (mm)	0.35×0.16×0.10	0.35×0.16×0.10	0.35×0.16×0.10
crystal system	orthorhombic	orthorhombic	orthorhombic
space group, *Z, Z'*	$P2_12_12_1$, 4, 1	$P2_12_12_1$, 4, 1	$P2_12_12_1$, 4, 1
a (Å)	4.06406(18)	4.03673(14)	4.00341(11)
b (Å)	13.5231(6)	13.4739(4)	13.4119(3)
c (Å)	14.1996(6)	14.1499(5)	14.0916(4)
V (Å^3)	780.39(6)	769.62(4)	756.63(3)
ρ (g/cm^3)	2.679	2.717	2.763
μ (mm^{-1})	15.413	15.629	15.897
θ range (deg)	2.87 - 26.00	2.88 - 25.95	2.89 - 26.00
index ranges	$-5 \le h \le 5$	$-4 \le h \le 4$	$-4 \le h \le 4$
	$-16 \le k \le 16$	$-16 \le k \le 16$	$-16 \le k \le 16$
	$-17 \le l \le 17$	$-17 \le l \le 17$	$-17 \le l \le 17$
reflns collected	10906	10781	10543
R_{int}	0.0673	0.0554	0.0487
data [$I > 2\sigma(I)$]	1297	1370	1395
data/parameters	1520/84	1496/84	1476/84
GOF on F^2	1.000	0.995	1.011
R_1 [$I > 2\sigma(I)$]	0.0296	0.0216	0.0184
R_1 (all data)	0.0414	0.0264	0.0213
wR_2 (all data)	0.0563	0.0370	0.0357
lrgst diff peak (e/Å^3)	0.486	0.350	0.344
lrgst diff hole (e/Å^3)	–0.460	–0.302	–0.369
CCDC	2041515	2041516	2041517

Table S4 Selected bond lengths (Å) and bond angles (°) for 123TBB at 270, 200 and 100 K.

temperature (K)	270	200	100	temperature (K)	270	200	100
C11–Br11	1.889(5)	1.882(5)	1.888(4)	C11–C12–Br12	121.1(4)	121.0(4)	121.1(3)
C12–Br12	1.892(5)	1.888(4)	1.890(4)	C12–C11–Br11	121.4(4)	121.4(4)	121.2(3)
C13–Br13	1.873(6)	1.876(5)	1.889(4)	C12–C13–Br13	122.3(4)	121.4(4)	121.2(3)
C21–Br21	1.882(6)	1.883(5)	1.891(4)	C13–C12–Br12	119.4(4)	120.0(4)	120.3(3)
C22–Br22	1.888(5)	1.872(5)	1.886(4)	C14–C13–Br13	118.1(5)	118.6(4)	118.0(3)
C23–Br23	1.888(6)	1.881(5)	1.893(4)	C16–C11–Br11	117.1(5)	117.6(4)	117.8(3)
C11–C12	1.368(7)	1.384(7)	1.384(6)	C21–C22–Br22	120.7(4)	120.7(4)	120.7(3)
C11–C16	1.375(8)	1.382(7)	1.386(6)	C22–C21–Br21	121.6(4)	121.4(4)	121.4(3)
C12–C13	1.376(7)	1.391(6)	1.388(6)	C22–C23–Br23	121.2(4)	120.6(4)	120.6(3)
C13–C14	1.382(7)	1.367(7)	1.378(6)	C23–C22–Br22	120.5(4)	121.2(4)	121.1(3)
C14–C15	1.370(9)	1.378(8)	1.379(6)	C24–C23–Br23	117.6(4)	117.8(4)	117.8(3)
C15–C16	1.376(7)	1.382(7)	1.384(6)	C26–C21–Br21	117.6(5)	117.8(4)	117.7(3)
C21–C22	1.383(8)	1.400(7)	1.395(6)	C11–C12–C13	119.5(5)	119.0(4)	118.6(4)
C21–C26	1.373(7)	1.376(7)	1.384(6)	C11–C16–C15	118.9(6)	119.0(5)	119.6(4)
C22–C23	1.377(7)	1.390(6)	1.386(6)	C12–C11–C16	121.5(5)	121.1(5)	121.0(4)
C23–C24	1.369(7)	1.376(7)	1.370(6)	C12–C13–C14	119.5(6)	120.0(5)	120.8(4)
C24–C25	1.381(8)	1.387(7)	1.394(6)	C13–C14–C15	120.4(6)	120.7(5)	120.2(4)
C25–C26	1.372(8)	1.372(7)	1.375(6)	C14–C15–C16	120.2(6)	120.2(5)	119.9(4)
				C21–C22–C23	118.8(5)	118.0(4)	118.2(4)
				C21–C26–C25	119.5(6)	119.9(5)	119.6(4)
				C22–C21–C26	120.8(5)	120.7(5)	120.9(4)
				C22–C23–C24	121.2(6)	121.5(5)	121.6(4)
				C23–C24–C25	119.1(6)	119.0(5)	119.3(4)
				C24–C25–C26	120.7(6)	120.8(5)	120.4(4)

Table S5 Selected bond lengths (Å) and bond angles (°) for 124TBB at 270, 200 and 100 K.

temperature (K)	270	200	100	temperature (K)	270	200	100
C11–Br11	1.85(2)	1.861(19)	1.866(17)	C11–C12–Br12	117.6(17)	121.6(15)	120.2(13)
C12–Br12	1.88(2)	1.882(17)	1.896(17)	C12–C11–Br11	125.9(17)	123.7(15)	123.9(13)
C14–Br14	1.90(2)	1.91(2)	1.908(18)	C13–C12–Br12	119.8(18)	117.1(15)	117.6(14)
C21–Br21	1.90(2)	1.887(19)	1.883(18)	C13–C14–Br14	116.1(18)	117.7(16)	117.2(15)
C22–Br22	1.89(2)	1.882(17)	1.884(18)	C15–C14–Br14	119.5(16)	121.2(16)	120.0(14)
C24–Br24	1.88(2)	1.875(19)	1.882(18)	C16–C11–Br11	117.2(18)	116.8(15)	117.2(14)
C31–Br31	1.89(2)	1.908(19)	1.898(18)	C21–C22–Br22	120.9(16)	119.8(14)	120.1(14)
C32–Br32	1.88(2)	1.885(17)	1.896(17)	C22–C21–Br21	121.6(16)	121.7(14)	122.7(14)
C34–Br34	1.87(2)	1.910(18)	1.906(18)	C23–C22–Br22	119.0(18)	121.6(16)	119.4(15)
C11–C12	1.40(3)	1.36(3)	1.38(2)	C23–C24–Br24	119.5(17)	119.9(15)	120.6(14)
C11–C16	1.41(3)	1.42(2)	1.43(2)	C25–C24–Br24	119.4(16)	120.0(15)	119.3(14)
C12–C13	1.39(3)	1.40(3)	1.38(2)	C26–C21–Br21	118.2(17)	120.3(15)	118.9(14)
C13–C14	1.36(3)	1.39(2)	1.39(2)	C31–C32–Br22	123.5(18)	124.9(15)	123.0(14)
C14–C15	1.39(3)	1.36(3)	1.35(2)	C32–C31–Br31	120.7(17)	119.5(14)	121.1(14)
C15–C16	1.40(3)	1.38(3)	1.42(2)	C33–C32–Br32	116.9(17)	115.5(14)	116.1(14)
C21–C22	1.39(3)	1.44(3)	1.40(2)	C33–C34–Br34	118.1(17)	117.5(15)	116.5(14)
C21–C26	1.38(3)	1.35(2)	1.39(2)	C35–C34–Br34	121.6(17)	118.7(14)	119.3(13)
C22–C23	1.35(3)	1.35(2)	1.38(2)	C36–C31–Br31	119.4(18)	119.4(15)	117.9(14)
C23–C24	1.40(3)	1.37(2)	1.37(2)	C11–C12–C13	122.5(19)	121.1(17)	122.1(16)
C24–C25	1.36(3)	1.37(3)	1.41(3)	C11–C16–C15	122(2)	119.3(19)	118.2(17)
C25–C26	1.40(3)	1.40(2)	1.37(2)	C12–C11–C16	117(2)	119.5(18)	118.9(16)
C31–C32	1.36(3)	1.35(2)	1.36(2)	C12–C13–C14	118(2)	118.8(19)	118.0(18)
C31–C36	1.37(3)	1.37(2)	1.37(2)	C13–C14–C15	124(2)	120.9(19)	122.7(18)
C32–C33	1.40(3)	1.44(2)	1.43(2)	C14–C15–C16	117(2)	120.3(19)	120.0(17)
C33–C34	1.41(3)	1.38(2)	1.39(2)	C21–C22–C23	120.1(19)	118.6(17)	120.4(17)
C34–C35	1.35(3)	1.36(3)	1.36(2)	C21–C26–C25	119(2)	122.2(19)	121.7(18)
C35–C36	1.38(3)	1.38(3)	1.42(2)	C22–C21–C26	120(2)	118.0(18)	118.3(17)
				C22–C23–C24	120(2)	122.2(19)	120.6(18)
				C23–C24–C25	121(2)	120.1(19)	120.1(17)
				C24–C25–C26	119(2)	118.8(19)	118.8(18)
				C31–C32–C33	119(2)	119.6(17)	121.0(17)
				C31–C36–C35	122(2)	121.6(19)	120.4(18)
				C32–C31–C36	120(2)	121.0(19)	121.0(17)
				C32–C33–C34	119(2)	116.6(19)	116.0(18)
				C33–C34–C35	120(2)	123.7(18)	124.2(18)
				C34–C35–C36	119(2)	117.4(18)	117.5(17)

Table S6 Selected bond lengths (Å) and bond angles (°) for 135TBB at 270, 200 and 100 K.

temperature (K)	270	200	100	temperature (K)	270	200	100
C11–Br11	1.898(7)	1.894(5)	1.898(5)	C12–C11–Br11	118.9(5)	118.7(4)	118.9(4)
C13–Br13	1.891(6)	1.894(5)	1.900(4)	C16–C11–Br11	118.4(5)	118.6(4)	118.6(4)
C15–Br15	1.883(7)	1.889(5)	1.896(5)	C12–C13–Br13	118.9(5)	118.4(4)	118.3(3)
C11–C12	1.371(9)	1.382(7)	1.382(7)	C14–C13–Br13	119.1(5)	119.2(4)	118.7(4)
C11–C16	1.376(9)	1.376(7)	1.383(6)	C14–C15–Br15	119.9(6)	119.2(4)	119.3(4)
C12–C13	1.375(9)	1.383(7)	1.377(7)	C16–C15–Br15	118.4(5)	118.8(4)	118.3(4)
C13–C14	1.371(9)	1.374(7)	1.376(7)	C11–C12–C13	118.0(6)	117.5(4)	117.5(4)
C14–C15	1.380(10)	1.388(7)	1.386(7)	C11–C16–C15	117.4(6)	117.6(4)	117.4(4)
C15–C16	1.388(9)	1.386(7)	1.387(7)	C12–C11–C16	122.7(6)	122.7(5)	122.5(4)
				C12–C13–C14	122.0(6)	122.4(4)	123.0(4)
				C13–C14–C15	118.3(7)	117.9(5)	117.3(5)
				C14–C15–C16	121.6(7)	121.9(5)	122.3(5)

Table S7 Shortest intermolecular contacts (Å, °) for 123TBB at 270 and 200 K, compared to those commensurate with the sums of the van der Waals radii of respective atoms at 100 K (Bondi, 1964).

temperature (K)	270	200	100
Br11···Br12^{i}	3.6440(10)	3.6175(8)	3.5850(6)
C11–Br11···Br12^{i}	162.75(19)	162.83(17)	163.37(15)
Br11···Br12^{i}–C12^{i}	135.03(16)	135.19(14)	135.10(12)
C11–Br11···Br12^{i}–C12^{i}	89.0(6)	88.9(5)	89.1(5)
Br12···Br11^{ii}	3.6440(10)	3.6175(8)	3.5850(6)
C12–Br12···Br11^{ii}	135.03(16)	135.19(14)	135.10(12)
Br12···Br11^{ii}–C11^{ii}	162.75(19)	162.83(17)	163.37(15)
C12–Br12···Br11^{ii}–C11^{ii}	89.0(6)	88.9(5)	89.1(5)
Br13···C22^{iii}	3.593(6)	3.575(5)	3.548(4)
C13···Br13–C22^{iii}	130.7(2)	130.38(17)	129.91(14)
Br13···C22^{iii}–C23^{iii}	73.4(3)	73.2(3)	72.3(2)
C13–Br13···C22^{iii}–C23^{iii}	–145.7(4)	–146.2(3)	–147.2(3)
C22···Br13^{iv}	3.593(6)	3.575(5)	3.548(4)
C23–C22···Br13^{iv}	73.4(3)	73.2(3)	72.3(2)
C22···Br13^{iv}–C13^{iv}	130.7(2)	130.38(17)	129.91(14)
C23–C22···Br13^{iv}–C13^{iv}	–145.7(4)	–146.2(3)	–147.2(3)
Br13···C23^{iii}	3.460(6)	3.441(5)	3.393(4)
C13–Br13···C23^{iii}	147.3(2)	147.37(18)	147.34(15)
Br13···C23^{iii}–C24^{iii}	84.4(4)	84.0(3)	83.9(3)
C13–Br13···C23^{iii}–C24^{iii}	174.5(4)	174.5(4)	173.0(3)
C23···Br13^{iv}	3.460(6)	3.441(5)	3.393(4)
C24–C23···Br13^{iv}	84.4(4)	84.0(3)	83.9(3)
C23···Br13^{iv}–C13^{iv}	147.3(2)	147.37(18)	147.34(15)
C24–C23···Br13^{iv}–C1^{iv}	174.5(4)	174.5(4)	173.0(3)
Br13···C24^{iii}	3.595(6)	3.570(5)	3.522(4)
C13–Br13···C24^{iii}	169.3(2)	169.60(18)	169.58(15)
Br13···C24^{iii}–C25^{iii}	89.2(4)	89.0(3)	89.7(3)
C13–Br13···C24^{iii}–C25^{iii}	105(1)	104(1)	99.5(9)
C24···Br13^{iv}	3.595(6)	3.570(5)	3.522(4)
C25–C24···Br13^{iv}	89.2(4)	89.0(3)	89.7(3)
C24···Br13^{iv}–C13^{iv}	169.3(2)	169.6(2)	169.58(15)
C25–C24···Br13^{iv}–C13^{iv}	105(1)	104(1)	99.5(9)
Br23···C13	3.468(6)	3.439(5)	3.395(5)
C23–Br23···C13	142.42(19)	142.47(17)	142.66(14)
Br23···C13–C14	80.5(4)	80.4(3)	80.4(3)
C23–Br23···C13–C14	–165.9(4)	–165.9(4)	–165.6(3)
Br23···C14	3.514(6)	3.481(5)	3.443(4)
C23–Br23···C14	163.7(2)	163.73(17)	164.22(14)
Br23···C14–C15	92.7(4)	92.6(4)	92.8(3)
C23–Br23···C14–C15	–88.6(8)	–89.1(7)	–86.6(6)

Symmetry codes: (i) $-x + 1, y + 1/2, -z + 1/2$; (ii) $-x + 1, y - 1/2, -z + 1/2$; (iii) $x, y - 1, z$; (iv) $x, y + 1, z$.

Table S8 Shortest intermolecular contacts (Å, °) for 124TBB at 270 and 200 K, compared to those commensurate with the sums of the van der Waals radii of respective atoms at 100 K (Bondi, 1964).

temperature (K)	270	200	100
Br11···Br34^{i}	3.687(4)	3.651(3)	3.606(3)
C11–Br11···Br34^{i}	99.2(7)	99.4(6)	99.7(5)
Br11···Br34^{i}–C34^{i}	171.2(7)	172.6(6)	172.2(6)
C11–Br11···Br34^{i}–C34^{i}	156(5)	164(5)	164(4)
Br34···Br11^{ii}	3.687(4)	3.651(3)	3.606(3)
C34–Br34···Br11^{ii}	171.2(7)	172.6(6)	172.2(6)
Br34···Br11^{ii}–C11^{ii}	99.2(7)	99.4(6)	99.7(5)
C34–Br34···Br11^{ii}–C11^{ii}	156(5)	164(5)	164(4)
Br14···Br21^{iii}	3.751(4)	3.722(3)	3.676(3)
C14–Br14···Br21^{iii}	86.3(6)	86.2(6)	86.0(6)
Br14···Br21^{iii}–C21^{iii}	161.4(7)	162.0(6)	162.1(6)
C14–Br14···Br21^{iii}–C21^{iii}	–41(2)	–41(2)	–37(2)
Br21···Br14^{iv}	3.751(4)	3.722(3)	3.676(3)
C21–Br21···Br14^{iv}	161.4(7)	162.0(6)	162.1(6)
Br21···Br14^{iv}–C14^{iv}	86.3(6)	86.2(6)	86.0(6)
C21–Br21···Br14^{iv}–C14^{iv}	–41(2)	–41(2)	–37(2)
Br14···Br31	3.698(4)	3.672(3)	3.640(3)
C14–Br14···Br31	165.7(8)	165.2(7)	165.4(6)
Br14···Br31–C31	105.6(7)	105.0(6)	105.5(6)
C14–Br14···Br31–C31	–101(3)	–102(3)	–100(2)
Br21···Br24^{v}	3.630(4)	3.606(3)	3.565(3)
C21–Br21···Br24^{v}	102.7(6)	102.3(6)	103.3(5)
Br21···Br24^{v}–C24^{v}	174.2(7)	174.9(6)	175.9(6)
C21–Br21···Br24^{v}–C24^{v}	127(7)	139(7)	140(8)
Br24···Br21^{vi}	3.630(4)	3.606(3)	3.565(3)
C24–Br24···Br21^{vi}	174.2(7)	174.9(6)	175.9(6)
Br24···Br21^{vi}–C21^{vi}	102.7(6)	102.3(6)	103.3(5)
C24–Br24···Br21^{vi}–C21^{vi}	–127(7)	–139(7)	–140(8)
Br12···H13^{vii}	2.96	3.01	2.97
C12–Br12···H13^{vii}	117	118	117
Br12···H13^{vii}–C13^{vii}	136	133	133
C12–Br12···H13^{vii}–C13^{vii}	–89	–92	–91
H13···Br12^{viii}	2.96	3.01	2.97
C13–H13···Br12^{viii}	136	133	133
H13···Br12^{viii}–C12^{viii}	117	118	117
C13–H13···Br12^{viii}–C12^{viii}	–89	–92	–91
Br12···H35^{ix}	3.07	3.05	3.00
C12–Br12···H35^{ix}	119	117	117
Br12···H35^{ix}–C35^{ix}	136	137	136
C12–Br12···H35^{ix}–C35^{ix}	162	167	167
H35···Br12^{x}	3.07	3.05	3.00
C35–H35···Br12^{x}	136	137	136
H35···Br12^{x}–C12^{x}	119	117	117
C35–H35···Br12^{x}–C12^{x}	162	167	167
Br22···H33^{xi}	3.14	3.07	3.04
C22–Br22···H33^{xi}	96	94	95
Br22···H33^{xi}–C33^{xi}	149	152	152
C22–Br22···H33^{xi}–C33^{xi}	141	147	145
H33···Br22^{xii}	3.14	3.07	3.04
C33···H33–Br22^{xii}	149	152	152
H33···Br22^{xii}–C22^{xii}	96	94	95
C33–H33···Br22^{xii}–C22^{xii}	141	147	145

Symmetry codes: (i) *x* – 1/2, *y*, *z* + 3/2; (ii) *x* + 1/2, *y*, *z* – 3/2; (iii) *x*, *y*, *z* + 1; (iv) *x*, *y*, *z* – 1; (v) *x* + 1/4, –*y* + 1/4, *z* – 3/4; (vi) *x* – 1/4, –*y* + 1/4, *z* + 3/4; (vii) –*x* + 1/2, –*y*, *z* + 1/2; (viii) –*x* + 1/2, –*y*, *z* – 1/2; (ix) –*x* + 1/2, –*y*, *z* + 3/2; (x) –*x* + 1/2, –*y*, *z* – 3/2; (xi) *x* – 1/2, *y*, *z* + 1/2; (xii) *x* + 1/2, *y*, *z* – 1/2.

Table S9 Hydrogen bonds geometries (Å, °) for 124TBB at 270, 200, compared to those commensurate with the sum of the van der Waals radii of Br and H at 100 K (Bondi, 1964).

	D–H	H···A	D···A	D–H···A
270 K				
C13–H13···Br12^{viii}	0.93	2.96	3.69(2)	137
C33–H33···Br22^{xii}	0.93	3.14	3.96(2)	149
C35–H35···Br12^{x}	0.93	3.07	3.80(2)	136
200 K				
C13–H13···Br12^{viii}	0.93	3.01	3.71(2)	133
C33–H33···Br22^{xii}	0.93	3.07	3.92(2)	152
C35–H35···Br12^{x}	0.93	3.05	3.78(2)	137
100 K				
C13–H13···Br12^{viii}	0.93	2.97	3.66(2)	133
C33–H33···Br22^{xii}	0.93	3.04	3.88(2)	152
C35–H35···Br12^{x}	0.93	3.00	3.723(19)	136

Symmetry codes: (viii) $-x + 1/2, -y, z - 1/2$; (x) $-x + 1/2, -y, z - 3/2$; (xii) $x + 1/2, y, z - 1/2$.

Table S10 Shortest intermolecular contacts (Å, °) for 135TBB at 270 and 200 K, compared to those commensurate with the sums of the van der Waals radii of respective atoms at 100 K (Bondi, 1964).

temperature (K)	270	200	100
Br13···Br13^{i}	3.7411(13)	3.7094(9)	3.6724(8)
C13–Br13···Br13^{i}	151.9(2)	152.15(16)	152.14(15)
Br13···Br13^{i}–C13^{i}	112.4(2)	112.16(15)	112.30(14)
C13–Br13···Br13^{i}–C13^{i}	–123.1(5)	–122.5(4)	–123.1(4)
Br13···Br13^{ii}	3.7411(13)	3.7094(9)	3.6724(8)
C13–Br13···Br13^{ii}	112.4(2)	112.16(15)	112.30(14)
Br13···Br13^{ii}–C13^{ii}	151.9(2)	152.15(16)	152.14(15)
C13–Br13···Br13^{ii}–C13^{ii}	–123.1(5)	–122.5(4)	–123.1(4)
Br13···Br15^{iii}	3.7458(11)	3.7252(8)	3.6994(7)
C13–Br13···Br15^{iii}	118.4(2)	117.95(15)	117.93(15)
Br13···Br15^{iii}–C15^{iii}	156.1(2)	155.94(17)	156.24(16)
C13–Br13···Br15^{iii}–C15^{iii}	–69.0(6)	–69.7(4)	–69.7(4)
Br15···Br13^{iv}	3.7458(11)	3.7252(8)	3.6994(7)
C15–Br15···Br13^{iv}	156.1(2)	155.94(17)	156.24(16)
Br15···Br13^{iv}–C13^{iv}	118.4(2)	117.95(15)	117.93(15)
C15–Br15···Br13^{iv}–C13^{iv}	–69.0(6)	–69.7(4)	–69.7(4)
Br13···C13^{v}	3.605(7)	3.572(5)	3.549(5)
C13–Br13···C13^{v}	89.8(2)	89.79(16)	89.25(15)
Br13···C13^{v}–C14^{v}	93.1(4)	93.7(3)	93.4(3)
C13–Br13···C13^{v}–C14^{v}	60.9(5)	60.8(4)	61.3(4)
C13···Br13^{vi}	3.605(7)	3.572(5)	3.549(5)
C14–C13···Br13^{vi}	93.1(4)	93.7(3)	93.4(3)
C13···Br13^{vi}–C13^{vi}	89.8(2)	89.79(16)	89.3(2)
C14–C13···Br13^{vi}–C13^{vi}	60.9(5)	60.8(4)	61.3(4)

Symmetry codes: (i) $x - 1/2, -y + 1/2, -z$; (ii) $x + 1/2, -y + 1/2, -z$; (iii) $-x + 1/2, -y + 1, z - 1/2$; (iv) $-x + 1/2, -y + 1, z + 1/2$; (v) $x - 1, y, z$; (vi) $x + 1, y, z$.

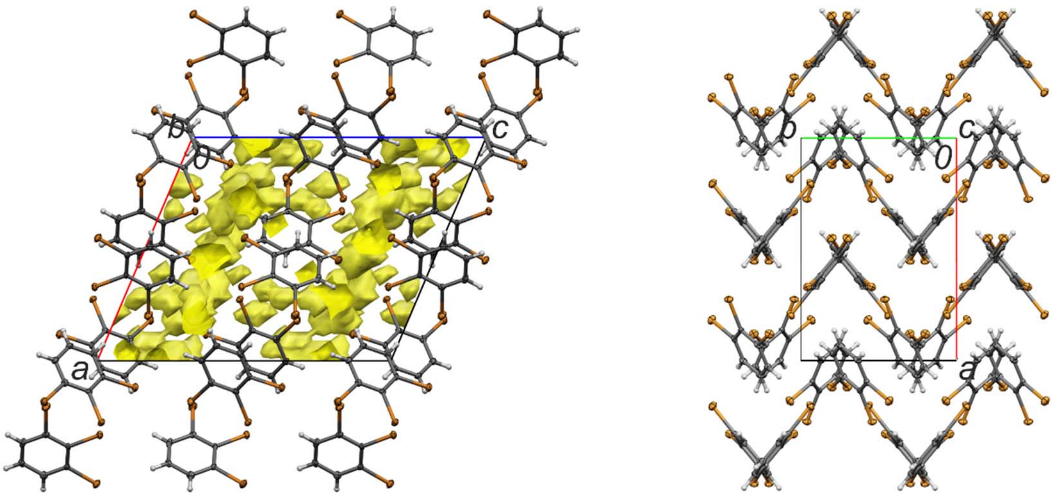

Figure S1 Structure of 123TBB, at 100 K, along (left) and perpendicular (right) to the shortest unit cell *b*-axis. The intermolecular space accessible to a probing sphere of radius 0.45 Å and grid spacing of 0.45 Å is indicated in yellow. The void volume is 8.6% (129.48 Å^3, Macrae *et al.*, 2020). Displacement ellipsoids are plotted at the 50% probability level.

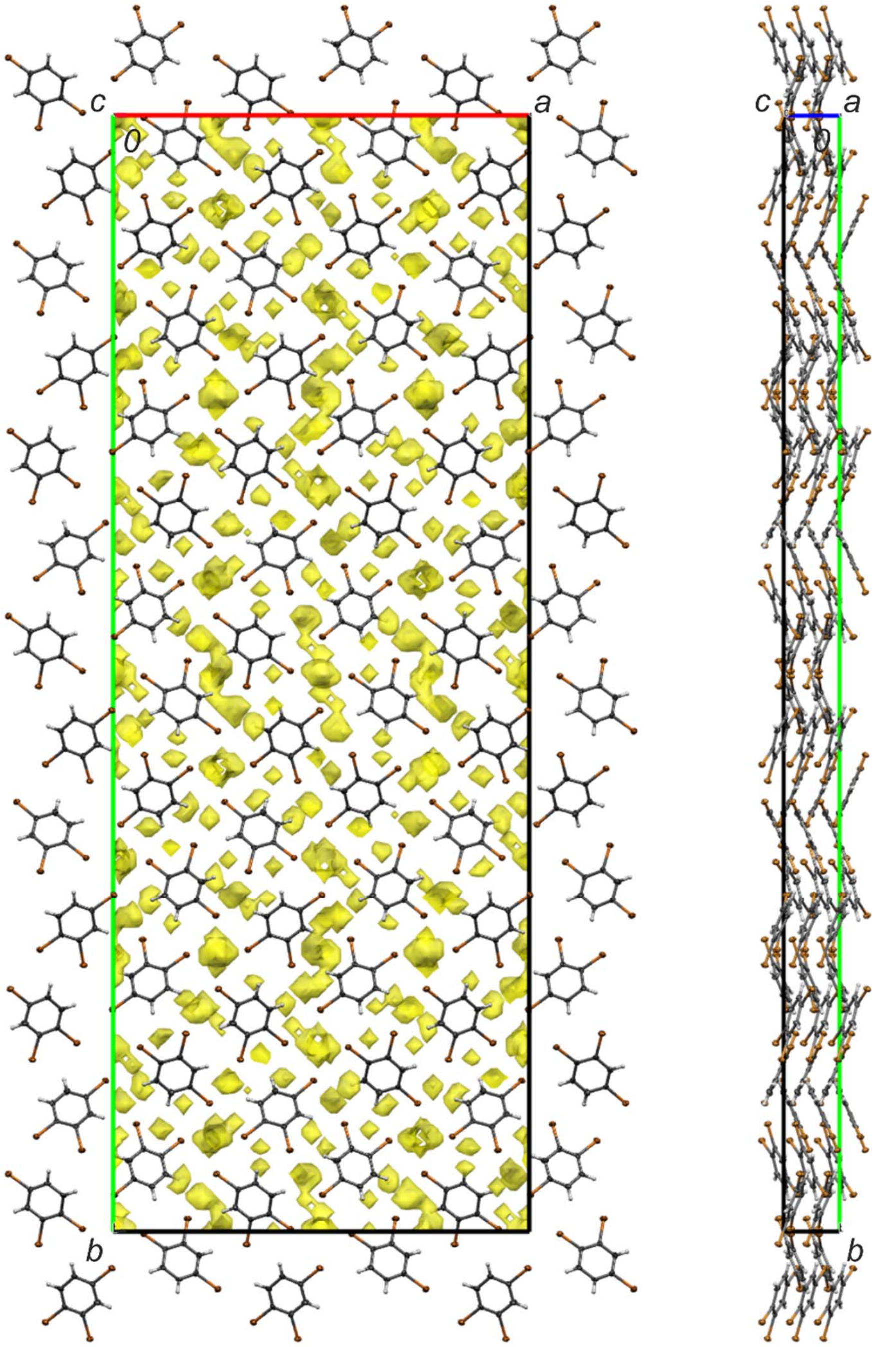

Figure S2 Structure of 124TBB, at 100 K, along (left) and perpendicular (right) to the shortest unit cell *c*-axis. The intermolecular space accessible to a probing sphere of radius 0.45 Å and grid spacing of 0.45 Å is indicated in yellow. The void volume is 5.4% (492.51 Å^3, Macrae *et al.*, 2020). Displacement ellipsoids are plotted at the 50% probability level.

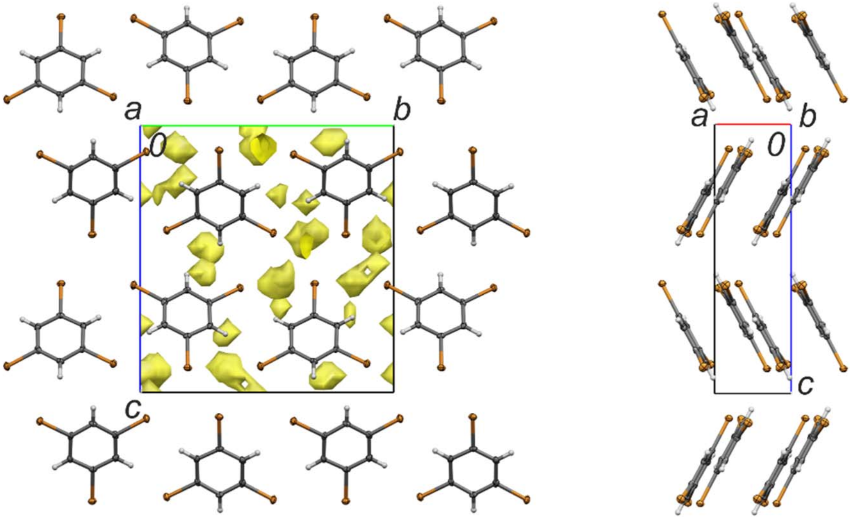

Figure S3 Structure of 135TBB, at 100 K, along (left) and perpendicular (right) to the shortest unit cell *a*-axis. The intermolecular space accessible to a probing sphere of radius 0.45 Å and grid spacing of 0.45 Å is indicated in yellow. The void volume is 3.9% (29.42 Å^3, Macrae *et al.*, 2020). Displacement ellipsoids are plotted at the 50% probability level.

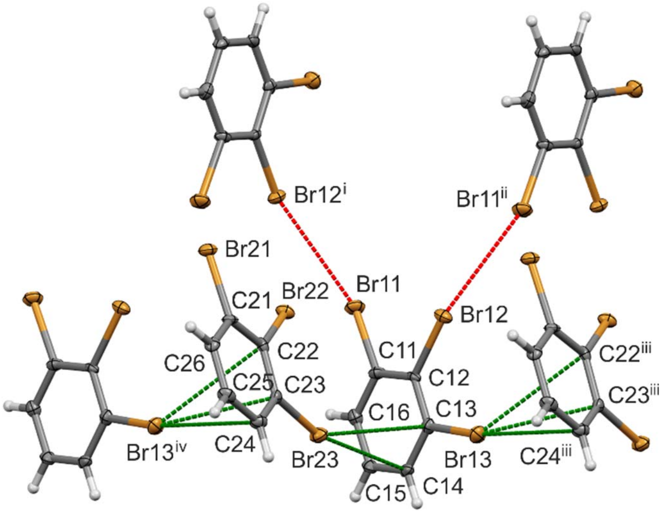

Figure S4 Symmetry-independent molecules along with their environment, at 100 K, in 123TBB. The broken red and green lines represent Br···Br and Br···C interactions, respectively, formed by these molecules (see Table S7). Displacement ellipsoids are plotted at the 50% probability level. Symmetry codes: (i) $-x + 1, y + 1/2, -z + 1/2$; (ii) $-x + 1, y - 1/2, -z + 1/2$; (iii) $x, y - 1, z$; (iv) $x, y + 1, z$.

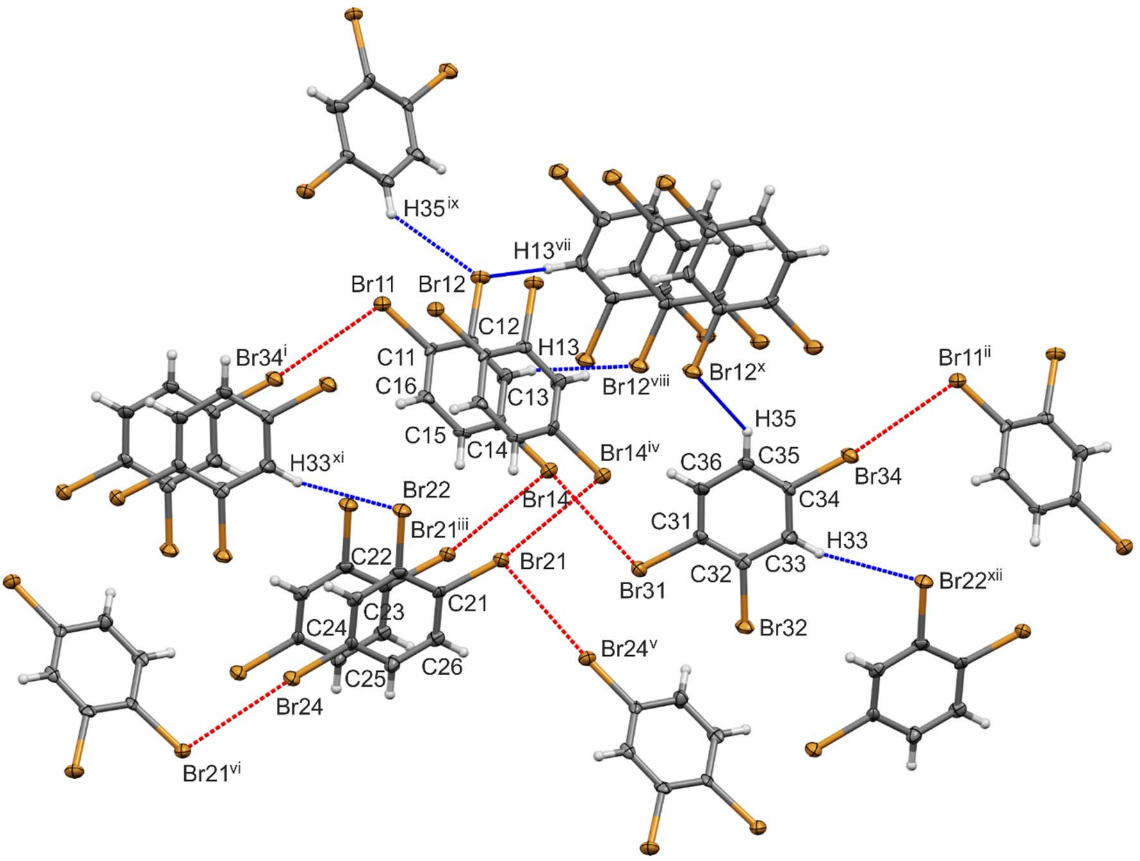

Figure S5 Symmetry-independent molecules along with their environment, at 100 K, in 124TBB. The broken red and blue lines represent Br···Br and Br···H interactions, respectively, formed by these molecules (see Tables S8 and S9). Displacement ellipsoids are plotted at the 50% probability level. Symmetry codes: (i) *x* – 1/2, *y*, *z* + 3/2; (ii) *x* + 1/2, *y*, *z* – 3/2; (iii) *x*, *y*, *z* + 1; (iv) *x*, *y*, *z* – 1; (v) *x* + 1/4, –*y* + 1/4, *z* – 3/4; (vi) *x* – 1/4, –*y* + 1/4, *z* + 3/4; (vii) –*x* + 1/2, –*y*, *z* + 1/2; (viii) –*x* + 1/2, –*y*, *z* – 1/2; (ix) –*x* + 1/2, –*y*, *z* + 3/2; (x) –*x* + 1/2, –*y*, *z* – 3/2; (xi) *x* – 1/2, *y*, *z* + 1/2; (xii) *x* + 1/2, *y*, *z* – 1/2.

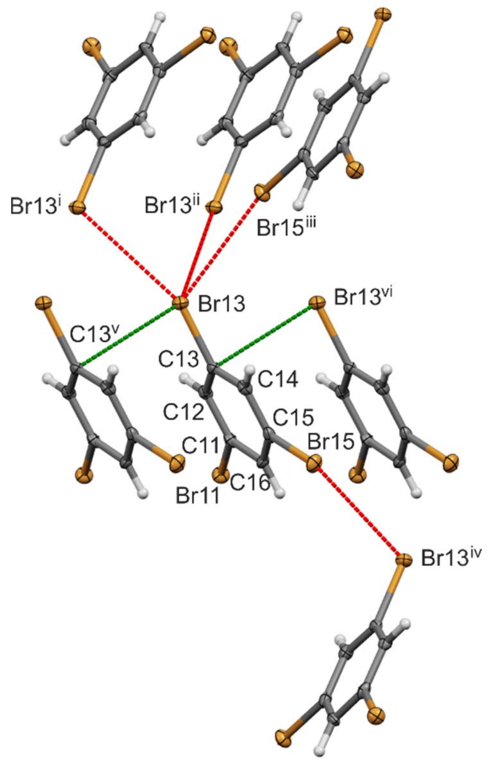

Figure S6 Symmetry-independent molecule along with its environment, at 100 K, in 135TBB. The broken red and green lines represent Br···Br and Br···C interactions, respectively, formed by this molecule (see Table S10). Displacement ellipsoids are plotted at the 50% probability level. Symmetry codes: (i) $x - 1/2, -y + 1/2, -z$; (ii) $x + 1/2, -y + 1/2, -z$; (iii) $-x + 1/2, -y + 1, z - 1/2$; (iv) $-x + 1/2, -y + 1, z + 1/2$; (v) $x - 1, y, z$; (vi) $x + 1, y, z$.

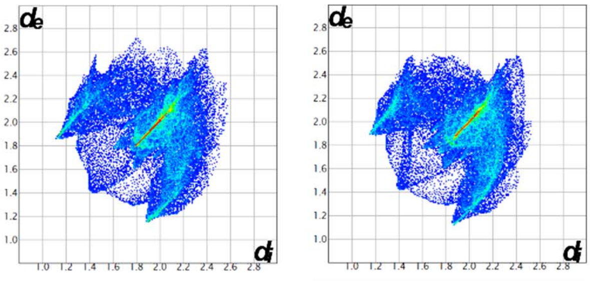

(a)

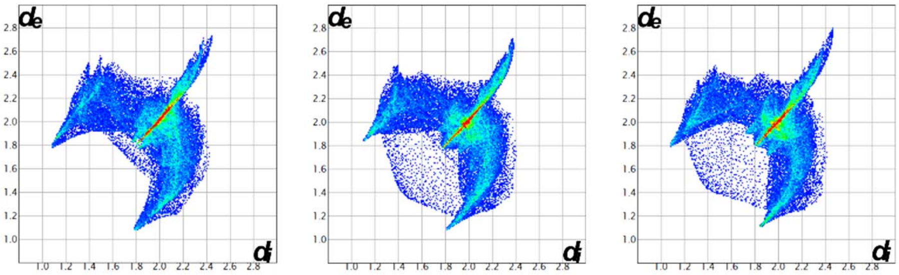

(b)

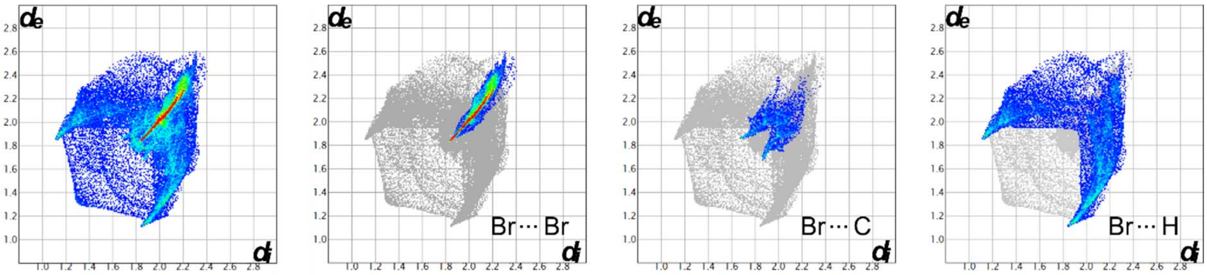

(c)

Figure S7 Fingerprints generated separately for symmetry-independent molecules at 100 K: (a) 123TBB molecules C(11-16) – left, C(21-26) – right; (b) 124TBB molecules C(11-16) – left, C(21-26) – middle; C(31-36) – right; and (c) that for 135TBB along with decomposed into the Br···Br, Br···C (Br···π) and Br···H contacts.

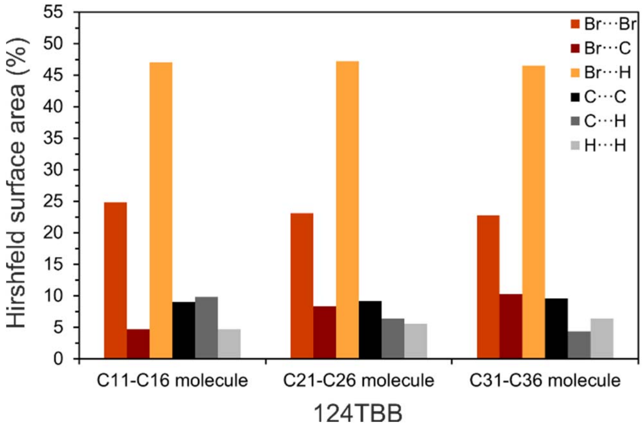

Figure S8 Distribution of contacts at 100 K, based on their area on Hirshfeld surfaces, for the C(11-16), C(21-26) and C(31-36) independent molecules in 124TBB.

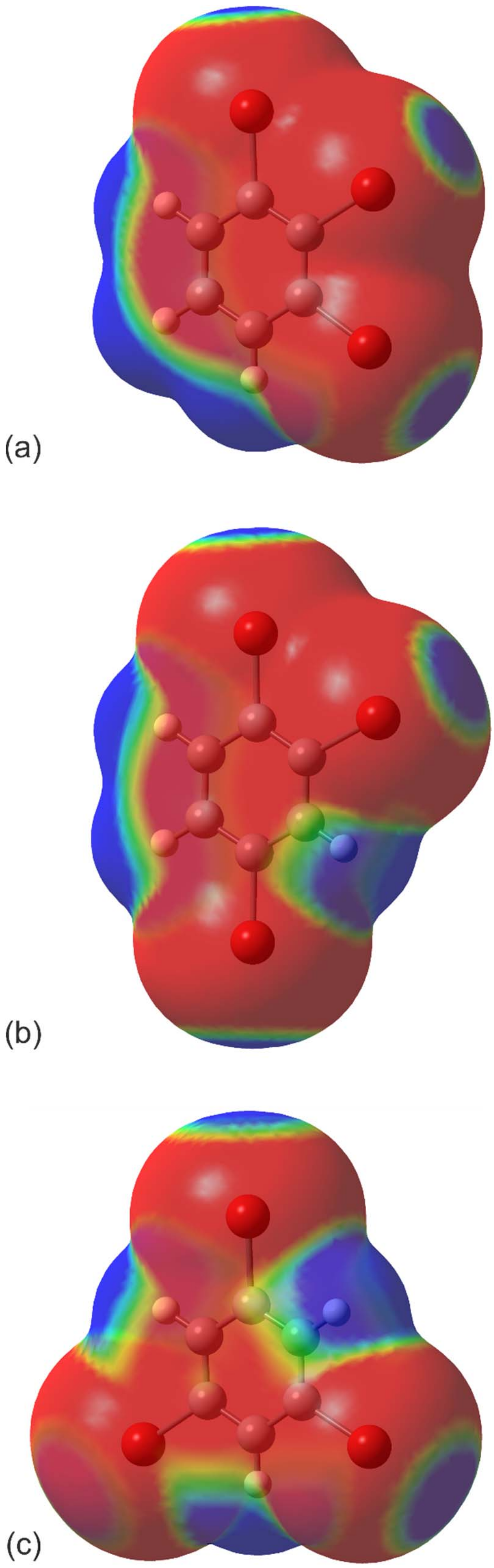

Figure S9 Molecular isodensity surfaces mapped with their electrostatic potential for: (a) 123TBB, (b) 124TBB and (c) 135TBB. The colours show positive from 0.01 a.u. (blue) to negative –0.001 a.u. (red) and intermediate degrees (orange-green) of electrostatic potential (Frisch *et al.*, 2009; Dennington *et al.*, 2009).

References (SI)

Bondi, A. (1964). *J. Phys. Chem.* **68**, 441–451.

Dennington, R., Keith, T. & Millam, J. (2009). *GaussView 5.0*, Semichem Inc., Shawnee Mission KS, USA.

Dziubek, K. & Katrusiak, A. (2014). *Acta Cryst.* B**70**, 492–497.

Frisch, M. J., Trucks, G. W., Schlegel, H. B., Scuseria, G. E., Robb, M. A., Cheeseman, J. R., Scalmani, G., Barone, V., Mennucci, B., Petersson, G. A., Nakatsuji, H., Caricato, M., Li, X., Hratchian, H. P., Izmaylov, A. F., Bloino, J., Zheng, G., Sonnenberg, J. L., Hada, M., Ehara, M., Toyota, K., Fukuda, R., Hasegawa, J., Ishida, M., Nakajima, T., Honda, Y., Kitao, O., Nakai, H., Vreven, T., Montgomery, Jr., J. A., Peralta, J. E., Ogliaro, F., Bearpark, M., Heyd, J. J., Brothers, E., Kudin, K. N., Staroverov, V. N., Kobayashi, R., Normand, J., Raghavachari, K., Rendell, A., Burant, J. C., Iyengar, S. S., Tomasi, J., Cossi, M., Rega, N., Millam, J. M., Klene, M., Knox, J. E., Cross, J. B., Bakken, V., Adamo, C., Jaramillo, J., Gomperts, R., Stratmann, R. E., Yazyev, O., Austin, A. J., Cammi, R., Pomelli, C., Ochterski, J. W., Martin, R. L., Morokuma, K., Zakrzewski, V. G., Voth, G. A., Salvador, P., Dannenberg, J. J., Dapprich, S., Daniels, A. D., Farkas, O., Foresman, J. B., Ortiz, J. V., Cioslowski, J. & Fox, D. J. (2009). *Gaussian 09*, Revision A.02, Gaussian, Inc., Wallingford CT, USA.

Kossiakoff, A. & Springall, H. D. (1941). *J. Am. Chem. Soc.* **63**, 2223–2230.

de Laszlo, H. (1934). *Proc. R. Soc. London, Ser. A*, **146**, 690–700.

Macrae, C. F., Sovago, I., Cottrell, S. J., Galek, P. T. A., McCabe, P., Pidcock, E., Platings, M., Shields, G. P., Stevens, J. S., Towler, M. & Wood, P. A. (2020). *J. Appl. Cryst.* **53**, 226–235.

Milledge, H. J. & Pant, L. M. (1960). *Acta Cryst.* **13**, 285–290.

Novikov, V. P., Sokolkov, S. V., Golubinskii, A. V. & Vilkov, L. V. (1986). *J. Struct. Chem.* **27**, 45–49.

Reddy, C. M., Kirchner, M. T., Gundakaram, R. C., Padmanabhan, K. A. & Desiraju, G. R. (2006). *Chem.-Eur. J.*, **12**, 2222–2234.

Saha, S. & Desiraju, G. R. (2017). *J. Am. Chem. Soc.* **139**, 1975–1983.

Woińska, M., Grabowsky, S., Dominiak, P. M., Woźniak, K. & Jayatilaka, D. (2016). *Sci. Adv.* **2**, e1600192.

Zakharov, B. A., Michalchuk, A. A. L., Morrison, C. A. & Boldyreva, E. V. (2018). *Phys. Chem. Chem. Phys.* **20**, 8523–8532.
